# COVID-19: an Immunopathological View

**DOI:** 10.1128/mSphere.00344-20

**Published:** 2020-04-22

**Authors:** Kamran Kadkhoda

**Affiliations:** a Immunopathology Laboratory, Robert J. Tomsich Pathology & Laboratory Medicine Institute, Cleveland Clinic, Cleveland, Ohio, USA; National Institute of Allergy and Infectious Diseases

**Keywords:** COVID-19, SARS-CoV-2, immunopathology

## Abstract

Since its emergence in December 2019, it took only a couple of months for an outbreak of the novel coronavirus disease 2019 (COVID-19) to be declared a pandemic by the World Health Organization (WHO). This along with the highly infectious nature of the disease and the associated mortality call for particular attention to the underlying (immuno)pathomechanism(s). The latter will inform case management and vaccine design. Unravelling these mechanisms can assist basic scientists, laboratory medicine practitioners, clinicians, public health practitioners, funding agencies, and health care policymakers in responding to the severe acute respiratory syndrome coronavirus 2 (SARS-CoV-2) pandemic.

## PERSPECTIVE

Xu et al. (1) reported on a 50-year-old man with no known underlying conditions who presented with pneumonia. Despite the presence of lymphopenia and absence of neutropenia, all inflammatory cells observed on the lung biopsy specimen were mononuclear cells. Peripheral blood lymphocytes showed high levels of activation markers (HLA-DR and CD38) by flow cytometry. These cells were strongly perforin- and/or granulysin-positive CD8^+^ T cells or were inflammatory Th17 cells. This had led to significant damage to the lung tissues as demonstrated by diffuse alveolar damage, indicating acute respiratory distress syndrome (ARDS). This is consistent with high-level surface expression of angiotensin-converting enzyme 2 (ACE2), the severe acute respiratory syndrome coronavirus 2 (SARS-CoV-2) receptor, on pneumocytes (2). Unfortunately, the patient passed away, but it is important to note that methylprednisolone was started on day 8 after the onset of symptoms, while the catastrophic ARDS cascade was already under way. Interestingly, inhalational alpha interferon (IFN-α) was also instituted along with methylprednisolone. Since IFN-α can boost antiviral immune responses, it might have further played a role in the tissue damage in this case. On a related note, previous studies with macaques using SARS-CoV had shown that older macaques had stronger innate immune responses (including those related to IFN-α) compared with younger macaques. In contrast, expression of IFN-β, as an anti-inflammatory cytokine, was reduced in older macaques (3). It has recently been shown that interleukin-6 (IL-6) was also expressed significantly higher in patients who succumbed to coronavirus disease 2019 (COVID-19) than in survivors (4). Observations like this case made by a number of front-line clinicians since the inception of the COVID-19 pandemic have led to pointing fingers at immunopathology as the potential main culprit.

There have been several studies including the large epidemiological joint report by WHO and China (5) stating that the case fatality rate (CFR) is exceedingly low in pediatric patients especially in the very young (CFR of 21.9% for individuals over 80 years of age versus 0% for individuals under 8 years of age). This is a striking finding that further suggests an immunopathological component to this observation. Another observation is the seroprevalence of community-acquired coronaviruses among adults is very high (90 to 100%) (6), but this is not necessarily the case in pediatric patients (7). Additionally, there are antigenic commonalities among coronaviruses (CoVs); for instance, similar to SARS-CoV-2, CoV-NL63 also uses ACE2 as the receptor (8). As individuals age, the chance of exposure to common community-acquired CoVs (229E, OC43, NL63, and HKU1) increases. As a consequence, with such background anamnesis, once individuals are exposed to novel (zoonotic) CoVs such as SARS-CoV-2, the fast and furious immune response does the damage (the original antigenic sin [OAS]). According to OAS, the initial antigen imprints an immune response so that subsequent exposure to related antigen(s) preferentially chooses the already-existing memory cells. The resultant response, although brisk and apparently strong (a high-titer IgG response would be faster than that of IgM), may be unduly insufficient and inappropriate to the point that it might be initially nonprotective. In the context of COVID-19, since ACE2 is highly expressed in the gastrointestinal (GI) tract (9), shedding the virus in the stool is prolonged (10); however, diarrhea is uncommon likely because virus-specific effector memory T cells typically home to the mucosal surfaces they had previously encountered with an infection with a common CoV, i.e., upper and lower respiratory tract. As a result, despite ARDS in the lungs, almost no significant intestinal damage occurs, if at all. To further confound matters, lungs also express high levels of CD32a (FcγRIIa, typically on alveolar macrophages), whereas GI tissues express almost no CD32a protein (The Human Protein Atlas [https://www.proteinatlas.org/] [accessed March 2020]). It has recently been shown that SARS-CoV and the Middle East respiratory syndrome (MERS)-CoV take advantage of non- or subneutralizing antibodies and enter cells via surface CD32a receptors (Trojan horse mechanism) (11, 12). Therefore, another even more biologically plausible pathomechanism is the so-called antibody-dependent enhancement (ADE). ADE has also been shown in infection with dengue viruses, Zika virus, Ebola virus, and human immunodeficiency virus. ADE is not only triggered by neutralizing antibodies but also by nonneutralizing antibodies. Additionally, antibody affinity plays an important role in ADE (11, 13). The most important pathomechanism in COVID-19, therefore, could be ADE in which CD32a plays the central role. CD32a is expressed on the surfaces of monocytes and macrophages among other cells such as alveolar macrophages, and once aggregated by several IgG molecules, it transduces its signal through the associated immunoreceptor tyrosine-based activation motif (ITAM). This results in the release of proinflammatory cytokines including IFN-γ, tumor necrosis factor alpha, interleukin-1 (IL-1), and IL-6 (14). This IL-6 overproduction is the justification for using IL-6 receptor antagonists such as tocilizumab in COVID-19 clinical trials. It should be noted, however, that activation of ITAM occurs through phosphorylation by certain members of Src family of tyrosine kinases such as Fyn and Syk. Interestingly, agents such as cerdulatinib have dual inhibitory function against Syk as well as against Janus kinase family members (such as JAK1/2) (15), the latter also play key roles in IL-6 receptor signaling (16).

To complete the picture, SARS-CoV nucleoprotein and RNA had previously been found within alveolar macrophages of nonsurvivors (17). These cells also express dendritic cell-specific intercellular adhesion molecule-grabbing nonintegrin (DC-SIGN; CD209 and also CD209L). These surface molecules have been reported to be coreceptors for SARS-CoV entry (18). In this way, alveolar macrophages can function as a local niche for the virus and thereby pass it on to other cells.

Findings similar to the aforementioned epidemiological finding have been made for MERS-CoV in which disease severity increases with age and severe disease has been uncommon among pediatric patients (19). Furthermore, along the lines of ADE, it has been known for decades that immunization of cats with spike protein of feline CoV leads to more severe future infections with feline CoV in cats (11). Therefore, ADE is perhaps more effective when the time intervals between CoV infections are just long enough so antibody titers drop to at least subneutralizing levels. This may explain why association of older age with severe COVID-19 is not always the rule. Infection of alveolar macrophages through ADE may explain their excessive activation and generation of local hyperinflammatory environment and the resultant systemic cytokine storm that may even fulfill criteria for secondary hemophagocytic lymphohistiocytosis and macrophage activation syndrome. The pivotal involvement of macrophages is further highlighted by the fact that very high levels of ferritin are seen in severe COVID-19 cases (20). Additionally, the fact that severe COVID-19 is associated with much higher viral load compared with mild cases further suggests that ADE could play an important role in severe cases (21). Another explanation for age-associated severity of COVID-19 may be attributed to C-reactive protein (CRP). It is known that CRP levels increase with age and that serum CRP levels in adults are higher than in children, with the highest median level observed in the elderly (22). Furthermore, it has been demonstrated that CD32a is the major biological receptor for CRP (23).

Furthermore, the course of COVID-19 is in general more severe in those with underlying conditions such as hypertension, poorly controlled diabetes mellitus, and cardiovascular disease among others (4). This may be attributed to the known increased expression of CD32a on monocytes and macrophages in these patients (24). However, it is a common assumption that immunocompromised patients are at increased risk of developing severe COVID-19. To challenge this assumption, a report from Italy (25) showed that 3 out of 111 liver transplant recipients who passed away due to COVID-19 had received their transplants 10 years prior to death. Interestingly, these patients were stable with very low trough concentrations of calcineurin inhibitors in plasma. They, however, were all male, older than 65 years, overweight, diabetic, and on treatment for hypertension. In contrast, in the same study, 3 out of 40 individuals who had recently received liver transplants and were on intense immunosuppression drugs (without the other risk factors mentioned for the former group) and who tested positive for SARS-CoV-2 experienced an uneventful course of COVID-19. The CFR in the former group was 3% in contrast to the overall CFR of ca. 10% in Italy. Abatement of IFN-α signaling by calcineurin inhibitors may explain these observations. IFN-α can also be largely produced as a result of CD32a activation (26). Similar observations in other immunocompromised patient populations with larger sample sizes will be paramount to further understand the role of immunopathology in COVID-19.

All in all, further corroborative research is urgently needed to find out why advanced age is a major risk factor for COVID-19 and whether immunopathomechanisms can potentially be harnessed early enough to prevent irreversible consequences. This may be ground-breaking and would not only change the current individual patient management but also potentially inform prudent vaccine design and recommendations as was the case with dengue virus vaccination. The implications of ADE will be immense if IgG serology is used to determine immune status for health care workers, as a positive result does not necessarily mean one is immune to COVID-19 (even with a test specificity of 100%). More importantly, ADE may cause harm if plasma from clinically resolved patients is used for treatment. A very careful balance between risks and benefits established through large multicenter randomized controlled trials will provide answers to all these questions.
